# Epidemiology of adolescent gaming disorder: A 6-year population-based longitudinal study (2019–2024)

**DOI:** 10.1192/j.eurpsy.2026.12232

**Published:** 2026-06-15

**Authors:** Katharina Busch, Hanna Wiedemann, Lisa Klamert, Kerstin Paschke

**Affiliations:** German Center for Addiction Research in Childhood and Adolescence (DZSKJ), University Medical Center Hamburg-Eppendorf (UKE), Hamburg, Germany

**Keywords:** adolescents, epidemiology, gaming disorder, ICD-11, longitudinal study

## Abstract

**Background:**

Gaming disorder (GD), i.e., persistent problematic gaming with clinically significant impairment, is a growing concern; however, estimates of GD in representative adolescent samples are limited. This study aimed to examine GD prevalence, incidence, persistence, and risk factors for different epidemiological parameters across 6 years, covering pre- to post-COVID-19 pandemic.

**Methods:**

Population-based longitudinal data, including 3358 German adolescents, were collected annually from 2019 to 2024 using online surveys. GD prevalence estimates were assessed by standardized International Classification of Diseases (ICD-11)-based self-report questionnaires. Between-wave persistence and incidence of GD, sociodemographic data, gaming time, and stress perception were measured. Risk factors for incidence and persistence of GD were examined using generalized-estimating equation (GEE) models.

**Results:**

GD prevalence doubled from 2.9% in 2019 to 6.5% in 2023 and remained above prepandemic levels in 2024 (3.9%). Males showed higher prevalence estimates compared to females across most waves, and prevalence peaked in 2023 (8%). For females, GD prevalence doubled between 2019 (1.7%) and 2020 (3.4%), peaked in 2022 (6.1%), and declined thereafter. The incidence of GD increased during the pandemic. According to GEE models, male sex, higher perceived stress, and longer gaming times were risk factors for GD incidence; older age and higher education were protective factors. The persistence of GD reached its peak in 2020 (36.2%). Significant risk factors for GD persistence were higher stress perception and longer gaming time.

**Conclusions:**

Prevalence and incidence rates indicate that GD continues to be a significant concern, and pandemic-related increases affected both males and females. Prevention and intervention programs should target identified risk groups.

## Introduction

Video gaming is highly popular among adolescents, with continuously increasing usage time and frequency. The trend is reflected both in an increase in gaming behavior and the rapid expansion of the video game market’s global revenue, which is estimated to amount to 564.27 billion U.S. dollars in 2026 [1]. Recent studies further demonstrate the widespread prevalence of gaming among young people worldwide. Namely, the *Health Behaviour in School-aged Children* (HBSC) study reported that one-third of adolescents (aged 11–15 years) from Europe, Central Asia, and Canada played video games daily [2]. In comparison, 27% of 15-year-olds in OECD countries reported playing video games for around 3 hours per day in 2021–2022 [3], and 71% of 12–19-year-old Germans played video games daily or multiple times a week in 2025 [4]. This highlights the increased relevance of the phenomenon.

While popular as a leisure activity, problematic dimensions of video gaming have received much attention in recent years, particularly among adolescents. Due to their stage of development, this age group is more susceptible to developing pathological gaming behavior [5–7] and thus constitutes a risk group. To facilitate the identification and classification of problematic/pathological gaming behaviors, two classification systems introduced definitions for problematic/pathological gaming behaviors. Internet gaming disorder (IGD), describing problematic gaming, was included as a condition warranting further study in the Appendix/Section III of the *Diagnostic and Statistical Manual of Mental Disorders, Fifth Edition* (DSM-5) in 2013 [8]. According to the DSM-5, five or more out of the nine criteria should be fulfilled within the last 12 months. The criteria are (1) preoccupation with gaming, (2) withdrawal when not gaming, (3) tolerance, (4) loss of control over gaming behavior, (5) neglecting other activities, (6) continuation of gaming despite problems, (7) deception, (8) escapism, and (9) negative consequences due to gaming. Gaming disorder (GD) was officially recognized as a clinical diagnosis by the World Health Organization (WHO) in the International Classification of Diseases 11th Revision (ICD-11; https://icd.who.int/en/) in 2018 and is defined by (1) impaired control over gaming, (2) increased priority given to gaming over other aspects in life, and (3) the continuation despite negative consequences [9]. This pattern must be severe enough to significantly impair personal, social, or educational functioning and should be present for at least 12 months in general.

Previous studies have compared the clinical utility and diagnostic validity of the DSM-5 and ICD-11 criteria for GD; however, consensus regarding their applicability and interpretation remains inconsistent. For example, Saunders et al. [10] reviewed the background and rationale for the inclusion of GD in ICD-11. They concluded that, compared to IGD as defined by the DSM-5, GD yields smaller prevalence estimates and represents a more conservative diagnostic framework, potentially because the ICD-11 criteria focus on three core clinical features of addiction in addition to functional impairment. In comparison, the DSM-5 includes a number of peripheral criteria (e.g., deception or withdrawal) next to the three main clinical criteria, although only five out of the nine criteria need to be met. Previous studies have found higher thresholds for GD compared to IGD [11], highlighting its clinical utility to avoid over-pathologization; however, stricter cutoffs may potentially neglect those with milder symptoms for treatment interventions [10]. Although both DSM-5 and ICD-11 criteria show diagnostic validity, most studies on adolescent gaming used DSM-5-based instruments, and studies applying ICD-11 criteria remain limited.

Varying prevalence estimates for pathological gaming have been reported in previous research, depending on country/cultural context, population, sampling time, assessment tool, and applied diagnostic criteria (i.e., DSM-5; ICD-11; other). Two recent meta-analyses that predominantly included studies applying DSM-5 criteria to assess problematic gaming reported pooled prevalence estimates of IGD of 8.6% and 8.8% among adolescents worldwide [12, 13]. Representative samples of German adolescents, in turn, have reported prevalence rates of problematic gaming of 1.2% up to 5.7% [14, 15]. During the COVID-19 pandemic, reviews report increases in IGD prevalence and video game use among adolescents in Asian, European, and Australian countries [16], while others highlight specific lockdown periods to be one significant factor [17]. Only a limited number of studies from Asia have applied ICD-11 criteria so far. For example, GD prevalence among Chinese adolescents was assessed using the Gaming Disorder Symptom Questionnaire (GDSQ-21) with a 2.27% prevalence [18], and in Japan, the prevalence of GD among the general young population was assessed using the GAMES test, showing estimates of 7.6% [19]. While reliable prevalence rates are essential for GD to assess the clinical relevance using official diagnostic criteria, data on representative, population-based samples from Europe are lacking.

In addition to reliable prevalence estimates of GD based on established diagnostic criteria, understanding the longitudinal development of GD, such as its persistence and incidence, is essential for informing public policy yet remains insufficiently addressed in research. In a recent meta-analysis by Sun et al. [20], the categorical stability or persistence of GD (i.e., meeting the threshold for problematic gaming/GD at baseline and follow-up) among adolescents was 43%–45% after 1 year. In comparison, another study reported an even lower 12-month IGD persistence of 14.4% among adolescents [21]. Incidence (i.e., no GD at T1 but above GD-threshold at T2) can provide information on trends of new cases of GD within a given timeframe. Liu et al. [22] found an IGD incidence of 7.7% among adolescents in China, comparable to the incidence estimates of 6%–11.7% reported in other studies [21, 23, 24].

Identification of risk factors for GD is essential for early detection and targeted prevention and intervention programs. Previous studies reported male sex to be a risk factor for problematic gaming [12, 25]. However, criticism of male bias in GD research highlights the need to focus on females and use representative rather than convenience samples [26]. Other risk factors playing a role in IGD development over time include poor academic performance, stress, and gaming time [12]. In addition, IGD has been linked to various mental health problems, including depression [27] and anxiety [28]. Specifically, longitudinal and review studies support that pre-existing psychopathology seems to function as a precursor to IGD [29–31], increasing the risk of developing pathological symptomatology. Other studies, at the same time, found that IGD worsened the mental health of some youths, suggesting a bidirectional relationship [32].

Aiming to provide valuable insights into the prevalence, incidence, persistence, and risk factors of GD as an official diagnosis based on ICD-11 criteria, the present study used representative data on the development of adolescent GD over six waves, including the time period of COVID-19, to provide valuable insights into temporal trends and uncover essential treatment needs in affected adolescents. In detail, the study aimed to (1) examine GD prevalence trends in different sex and age groups between 2019 and 2024, (2) examine between-wave incidence and persistence of GD, and (3) identify sociodemographic risk factors of incidence and persistence of GD in a representative sample of German adolescents.

## Methods

### Participants and procedure

Data were derived from a large population-based longitudinal online survey study on digital media use and mental health in German families (i.e., adolescents and respective parents) [33]. Data were collected annually from 2019 (W1) to 2024 (W6), covering the COVID-19 pandemic (W1: prepandemic; W2–W3: mid-pandemic; W4: end-pandemic; W5–W6: post-pandemic). Participants were recruited from the web-based forsa Omninet panel by the German Institute for Social Research and Statistical Analysis, *forsa [34].* The *forsa* database in general encompasses more than 100,000 German adults and adolescents aged 14 and above. For this study, participants were selected from a cluster of German adults aged 28 to 75 years based on sex, age, education, and region to ensure representativeness and were included only if they reported to have at least one child between 10 and 17 years. In cases where a household included multiple eligible children, the child with the most recently passed birthday was selected. Parents and children completed the annual surveys. To compensate for attrition, new families were added to the panel in each wave (see Supplementary Methods for further details on the participant recruitment). In total, 3358 adolescents participated across the six waves. Of these, 1586 (47.2%) participated once, and 1772 (52.8%) participated in multiple waves. A subset of 131 adolescents (3.9%) participated in all six waves. For this study, we used both cross-sectional (aged 10–17 years) and longitudinal wave-pair data (aged 10–23 years) from participating adolescents (see Supplementary Figure S1 for a flow chart). This study was approved by the Local Psychological Ethics Committee at the Center for Psychosocial Medicine of the University Medical Center Hamburg-Eppendorf (UKE) and complied with the Declaration of Helsinki.

### Measures

#### Gaming disorder

GD was assessed using the Gaming Disorder Scale for Adolescents (GADIS-A) [35]. The self-report scale includes 10 items with two factors measuring cognitive-behavioral symptoms (factor 1: four items) and negative consequences (factor 2: five items), and one additional time criterion assessing whether symptoms persisted for 12 months. Items for both factors were scored using Likert scales ranging from strongly disagree (0) to strongly agree (4). Higher scores indicated a more problematic use. The criteria for GD were fulfilled if adolescents had reached the cutoff values for both factors (factor 1: cutoff >9 points and factor 2: cutoff >5 points) and fulfilled the 12-month time criterion (cutoff fulfilled when “during longer periods” or “almost daily”), yielding a dichotomous outcome variable (0 = no GD, 1 = GD). The internal consistency was excellent, with Cronbach’s alpha values ranging between 0.89 and 0.94 across waves.

#### Average weekly gaming times

The average time spent gaming was calculated among adolescents who reported gaming at least once per week, based on self-reports for weekdays (school days) and weekends (nonschool days) separately. Average weekly gaming time (in hours) was then calculated using the following formula: [(weekday minutes × 5 + weekend minutes × 2) / 7] × (number of usage days per week)/60.

#### Perceived stress

The Perceived Stress Scale – 4-item short form (PSS-4) was used to assess adolescents’ psychological stress perception in the past month [36]. Sum scores ranged from 0 to 16, with higher scores indicating higher general stress. In this study, Cronbach’s alpha was modest, with values of 0.57 to 0.65 throughout all waves.

#### Sociodemographic factors

Sociodemographic factors included age, biological sex (female, male), place of residence (rural: <5000 residents, urban: ≥ 5000 residents), and self-reported educational level ([prospective] school leaving qualification). Educational level was categorized as low (no certificate, specialized school, or lower secondary education), middle (middle secondary education), and high (upper secondary (A-levels) or higher education/university or other educational degree). Age was grouped into early (aged 10–13 years), mid (aged 14–17 years), and late adolescence/emerging adulthood (aged 18–23 years) [37, 38]. The latter group was used for incidence and persistence analyses only.

### Data analysis

#### Data analyses

Analyses were performed using R (version 4.4.3) [39]. Only participants with valid responses on the item assessing gaming days per week were included. To handle missing data, multiple imputation (MI) by chained equations (MICE) was employed [40]. The proportion of missing values ranged between 0% and 28% for the variables of interest. Ten imputed datasets (*m* = 10) were specified, and results were pooled using Rubin’s rules [41]. Descriptive statistics (i.e., mean, standard deviation, and frequency) were used to summarize the sample characteristics. To estimate the prevalence of GD, the pooled weighted prevalence for each wave was calculated with the R package *modelbased* [42]. Prevalence estimates were calculated using representative data of adolescents aged 10 to 17 years. Population-based survey weights provided by *forsa* were applied to calculate representative estimates adjusted for sociodemographic characteristics of the adolescents (biological sex, age, region; based on data from the Federal Statistical Office of Germany). In addition, the analyses were stratified by biological sex and age group, including (1) biological sex and (2) the interaction between biological sex and age group using pooled estimates and contrasts. Pairwise comparisons assessed differences between the groups within each wave (see Supplementary Table S7), and *p*-values were corrected for multiple comparisons using the FDR method. Sensitivity analyses for GD prevalence using complete case analysis yielded similar results (see Supplementary Table S6).

To investigate longitudinal patterns of GD, only participants providing data for two consecutive waves (e.g., wave 1 (W1) – wave 2 (W2); wave 2 (W2) – wave 3 (W3)) were included in the analysis. Between-wave incidence and persistence rates were calculated for each wave pair using *modelbased* [42], yielding five estimates in total (W1 ➔ W2, W2 ➔ W3, W3 ➔ W4, W4 ➔ W5, W5 ➔ W6). Incidence was calculated as the proportion of participants who did not meet the cutoff for GD at T1 but met the cutoff at T2. Persistence was calculated as the proportion of participants who met the cutoff for GD at T1 and T2.

To explore possible risk factors associated with the incidence and persistence of GD, data from all six waves were used and grouped into five wave pairs (*n*_pairs_ = 3354). Across all wave pairs, 1553 participants were included: 131 (8.4%) contributed to all five wave pairs, 143 (9.2%) to four wave pairs, 246 (15.8%) to three wave pairs, 356 (22.9%) to two wave pairs, and 677 (43.6%) to one wave pair. Two pooled generalized-estimating equation (GEE) models examined the association between sociodemographic risk factors and between-wave incidence (model 1) and persistence of GD (model 2) while controlling for average weekly gaming time, perceived stress, and start wave using the R package *geepack* [43]. GEE models account for the dependency of observations resulting from repeated measurements within individuals. They were used with a binomial family and logit link to estimate population-averaged odds ratios (OR) and 95% confidence intervals for GD incidence and persistence after testing model assumptions. To select the working correlation structure, the first imputed dataset was fitted using different options (independence, exchangeable, AR(1)), and the resulting quasi-information criterion (QIC) values were compared. The selection of the best structure is based on the smallest QIC. Small differences (<2) indicated GEE model robustness to the choice of working correlation structure [44]. Hence, the exchangeable structure was selected (see Supplementary Table S1). Sensitivity analyses using an alternative correlation were conducted and yielded similar results (see Supplementary Tables S2 and S3).

## Results

### Sample characteristics

Weighted sociodemographic characteristics of the cross-sectional samples across all six waves are presented in Table 1. See Supplementary Table S4 for the weighted, nonimputed sociodemographic characteristics and Supplementary Table S5 for sociodemographic characteristics of the longitudinal wave pair data.

**Table 1.** Weighted sociodemographic characteristics of the participating adolescents Table 1. long description.

Variables	2019 *n* = 1,206	2020 *n* = 1,128	2021 *n* = 1,103	2022 *n* = 1,010	2023 *n* = 1,067	2024 *n* = 997
	% / M (SD)	% / M (SD)	% / M (SD)	% / M (SD)	% / M (SD)	% / M (SD)
**Sex**						
Females	48.1	48.0	47.9	48.9	48.6	48.4
Males	51.9	52.0	52.1	51.1	51.4	51.6
**Age**	13.6 (2.3)	13.5 (2.2)	13.5 (2.3)	13.5 (2.3)	13.6 (2.3)	13.45 (2.3)
10–13 years	48.7	49.0	49.1	50.3	49.3	49.7
14–17 years	51.4	51.0	50.9	49.7	50.7	50.3
**Education**						
Low	9.5	10.9	11.7	13.6	15.9	11.7
Medium	37.6	37.1	38.8	39.2	31.3	34.6
High	52.9	52.0	49.5	47.2	52.8	53.7
**Place of residence**						
Urban	84.0	83.0	81.4	79.4	79.5	79.9
Rural	16.0	17.0	18.6	20.6	20.6	20.1
**Stress level**						
**PSS–4 total score**	6.3 (2.9)	5.7 (3.1)	6.1 (2.9)	5.5 (3.0)	5.3 (2.9)	5.4 (2.3)
**Average weekly gaming time (hours/week)**	12.5 (14.4)	13.8 (15.1)	12.5 (13.3)	12.6 (13.3)	11.8 (11.8)	13.0 (15.1)
**GADIS-A total score**	7.4 (7.4)	6.7 (7.6)	6.7 (7.5)	8.0 (8.1)	7.2 (8.1)	7.1 (7.6)

*Note:* Weighted sociodemographic information based on pooled results across multiple imputed datasets (*m* = 10). Abbreviations: GADIS-A, gaming disorder scale for adolescents; M, mean; PSS-4, perceived stress scale – short form; SD, standard deviation.

### GD prevalence

**Figure 1.**
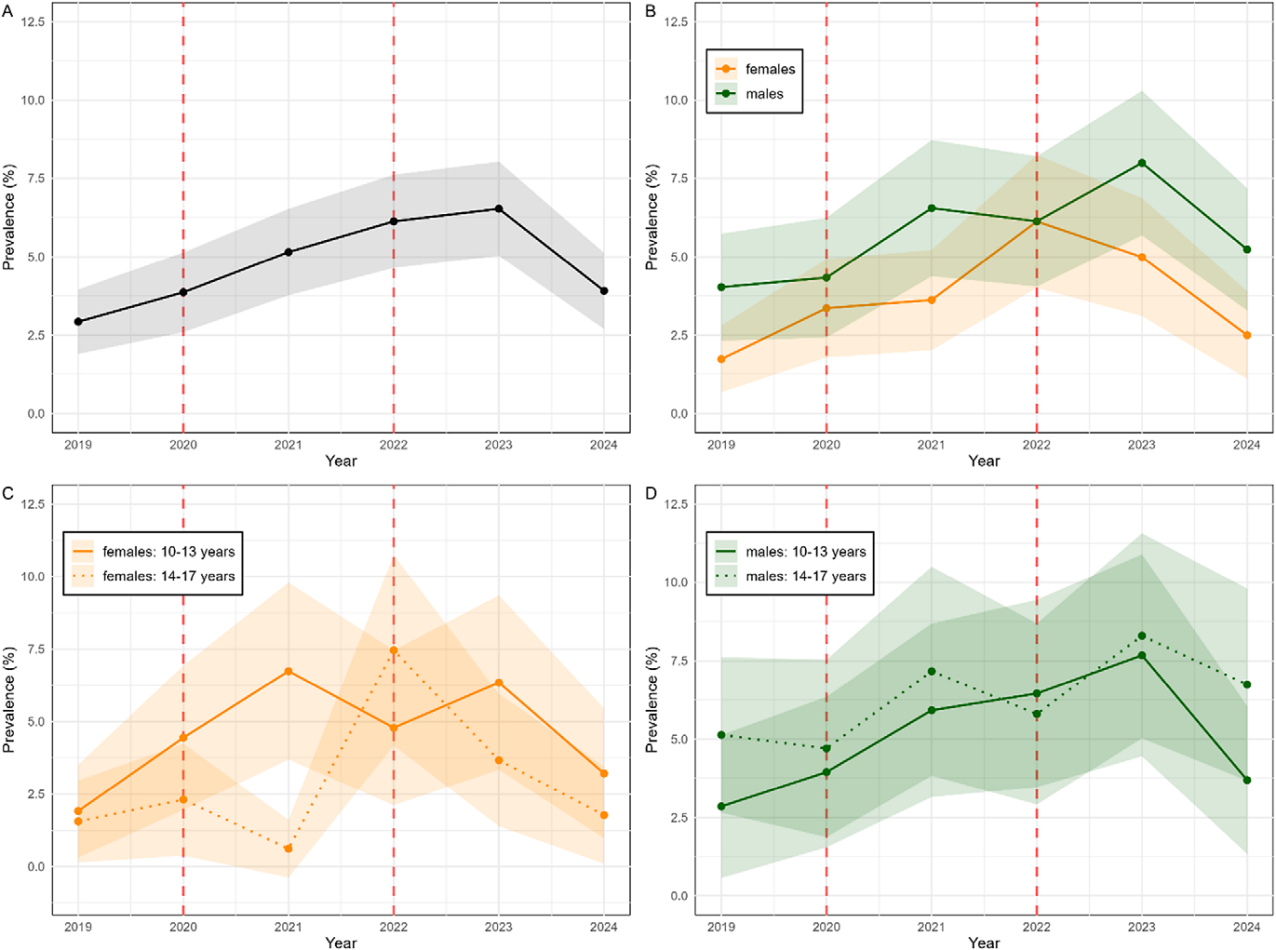
Trends of prevalence of GD based on ICD-11 criteria between 2019 and 2024. *Note*: Panel A shows overall GD prevalence estimates. Panel B shows GD prevalence estimates stratified by biological sex (females = orange; males = green). Panel C shows GD prevalence estimates of females stratified by age group (10–13 years = solid line; 14–17 years = dotted line). Panel D shows GD prevalence estimates of males stratified by age group (10–13 years = solid line; 14–17 years = dotted line). The red dotted line represents the COVID-19 pandemic. The ribbons represent 95% confidence intervals. Abbreviations: GD, gaming disorder; ICD-11, International Classification of Diseases 11th Revision. Figure 1. long description.

The prevalence estimate for GD was 2.9% in 2019 and peaked in 2023 at 6.5%. In 2024, the estimate remained above prepandemic levels at 3.91% (see Table 2 and Figure 1). Different trends for males and females were observed. In 2019, the prevalence estimate of GD was 4% among males and 1.7% among females (*p* = .075). With the start of the COVID-19 pandemic in 2020, the prevalence estimate for females doubled and increased slightly for males; the difference was not significant (*p* = .645). For males, the prevalence estimate continued to increase in 2021 (6.6%), slightly declined in 2022 (6.1%), and peaked at 8.0% in 2023. For females, the prevalence estimates also increased and differed significantly from males in 2021 (*p* = .048). In 2022, the prevalence for females peaked at 6.1%, reaching comparable values to males. In 2023, toward the end of the pandemic, the prevalence estimate for females decreased again but did not differ significantly from the estimate for males (*p* = .144). Comparably, the prevalence estimate for males decreased to 5.2% in 2024. Only in 2021, significant age group differences stratified by sex were found in our study, indicating that females aged 10 to 13 years compared to females aged 14 to 17 years showed significantly higher GD prevalence estimates (*p* < .001). In addition, descriptively, the prevalence of GD among adolescent females aged 10 to 13 years increased steadily until 2021 (6.7%), stayed elevated until 2023, and declined by 2024 (3.2%). In comparison, mid-adolescent females’ prevalence increased slightly by 2020, declined in 2021 but peaked in 2022 (7.5%), followed by a decline until 2024. On the one hand, for young males (10 to 13 years), the prevalence increased from 2.9% in 2019 to 5.9% in 2021, followed by a two-point increase and a peak in 2023 (7.7%). For males aged 14 to 17 years, on the other hand, GD prevalence was 5.1% in 2019, increased to 7.2% in 2021, slightly decreased again but peaked in 2023 with 8.3%. See Supplementary Table S7 for pairwise comparisons between sex and age group differences in GD prevalence within each wave.

**Table 2.** Weighted prevalence estimates of GD based on ICD-11 criteria between 2019 and 2024 Table 2. long description.

Groups	2019*n* = 1,206	2020*n* = 1,128	2021*n* = 1,103	2022*n* = 1,010	2023*n* = 1,067	2024*n* = 997
	Estimate %[95% CI]	Estimate %[95% CI]	Estimate %[95% CI]	Estimate %[95% CI]	Estimate %[95% CI]	Estimate %[95% CI]
**Total**	**2.92** [1.90;3.95]	**3.86** [2.59;5.13]	**5.15** [3.77;6.53]	**6.13** [4.64;7.61]	**6.53** [5.03;8.03]	**3.91** [2.70;5.12]
**Females**	1.74 [0.67;2.8]	3.36 [1.79;4.92]	3.62 [2.02;5.21]	6.13 [4.01;8.24]	4.99 [3.11;6.87]	2.50 [1.10;3.89]
10–13 years	1.92 [0.32;3.51]	4.45 [1.97;6.93]	6.74 [3.70;9.79]	4.79 [2.12;7.46]	6.35 [3.35;9.35]	3.22 [0.98;5.45]
14–17 years	1.56 [0.15;2.97]	2.31 [0.38;4.24]	0.62 [−0.37;1.6]	7.46 [4.18;10.74]	3.67 [1.40;5.95]	1.78 [0.11;3.45]
**Males**	4.03 [2.32;5.73]	4.33 [2.42;6.24]	6.55 [4.38;8.73]	6.13 [4.05;8.21]	7.99 [5.69;10.29]	5.23 [3.29;7.18]
10–13 years	2.85 [0.57;5.13]	3.95 [1.55;6.35]	5.92 [3.15;8.68]	6.45 [3.47;9.44]	7.67 [4.45;10.89]	3.69 [1.34;6.05]
14–17 years	5.13 [2.65;7.62]	4.70 [1.87;7.53]	7.17 [3.83;10.50]	5.80 [2.91;8.68]	8.30 [5.03;11.57]	6.74 [3.68;9.80]

*Note*: Weighted prevalence estimates are based on pooled results across multiple imputed datasets (*m* = 10). Results were combined using Rubin’s rules. Abbreviations: CI, confidence interval; GD, gaming disorder; ICD-11, International Classification of Diseases 11th revision. *n* = sample size.

### Incidence and persistence of GD

The pooled estimated between-wave incidence of GD increased during the COVID-19 pandemic from 1.7% in W1–W2 to 3.9% in W3–W4 and remained elevated post-pandemic at 2.6% in W5–W6 (see Table 3). The pooled estimated between-wave persistence of GD peaked in W1–W2 at 36.2% and stayed around 31% until W4–W5. In W5–W6, GD persistence decreased to 22.7%.

**Table 3.** Incidence and persistence of GD across wave pairs between 2019 and 2024 Table 3. long description.

Wave pair	Persistence of GD	Incidence of GD
	Estimate % [95% CI]	Estimate % [95% CI]
W1–W2	36.24 [12.25; 60.22]	1.69 [0.66; 2.72]
W2–W3	31.30 [5.97; 56.64]	3.44 [2.11; 4.78]
W3–W4	31.87 [15.47; 48.26]	3.89 [2.46; 5.33]
W4–W5	31.53 [17.24; 45.81]	3.56 [1.99; 5.13]
W5–W6	22.70 [8.14; 37.25]	2.60 [1.32; 3.88]

*Note*: Estimates of between-wave incidence and persistence of GD are based on pooled results across multiple imputed datasets (*m* = 10). Results were combined using Rubin’s rules. Abbreviations: CI, confidence interval; GD, gaming disorder, W, wave.

### GEE models with incidence and persistence of GD

Two separate GEE models were estimated to examine the associations of the predictors with GD incidence and persistence (see Table 4). Findings indicate that age, biological sex, school education, stress perception, start wave, and weekly gaming time were significantly associated with the incidence of GD, after accounting for within-subject correlations. For GD persistence, only stress perception, education level, and weekly gaming time were significant.

**Table 4.** GEE analysis for the incidence of GD and persistence of GD Table 4. long description.

Predictor	Incidence of GD OR [95% CI]	*p*	Persistence of GD OR [95% CI]	*p*
**Age**	0.76 [0.61, 0.94]	.035*	0.88 [0.63, 1.24]	.467
**Biological sex** (Ref: Females)	–	–	–	–
Males	1.78 [1.13, 2.79]	.017*	1.44 [0.56, 3.66]	.448
**Education** (Ref: Low)	–	–	–	–
Middle	0.93 [0.51; 1.67]	.801	0.73 [0.31, 1.76]	.485
High	0.40 [0.21; 0.77]	.007**	0.27 [0.10; 0.73]	.01*
**Residence** (Ref: Rural)	–	–	–	–
Urban	1.32 [0.75, 2.33]	.327	0.79 [0.31, 2.01]	.526
**Covariates**				
Perceived stress	1.41 [1.14, 1.75]	.002**	1.86 [1.42, 2.44]	.009**
Gaming time	1.37 [1.20, 1.56]	< .001***	1.63 [1.33, 1.99]	< .001***
Start wave (Ref: Wave 1)	–	–	–	–
Wave 2	2.29 [1.07; 4.90]	.034*	0.66 [0.19; 2.29]	.515
Wave 3	2.61 [1.26; 5.38]	.01*	1.65 [0.62; 4.41]	.319
Wave 4	2.23 [1.03; 4.82]	.041*	2.63 [0.97; 7.15]	.057
Wave 5	1.82 [0.80; 4.15]	.152	1.47 [0.46; 4.62]	.513

*Note*: Odds ratios (OR) and 95% confidence intervals (CI) are presented. Results are pooled across all imputations (*m* = 10) using Rubin’s Rules. Numeric variables were *z*-standardized. *n* = 3354 observations were included across five wave pairs. * *p* < .05, ** *p* < .01, *** *p* < .001. Abbreviations: CI, confidence interval; GD, gaming disorder; GEE, generalized-estimating equations; OR, odds ratios.

## Discussion

To our knowledge, this study is the first to examine GD prevalence, incidence, and persistence estimates within representative adolescent population-based samples over 6 years. The findings show that the GD prevalence among adolescents doubled between 2019 and 2023 and remains above prepandemic levels. The odds of having GD incidence were 1.78 times higher among males compared to females. Older age and higher education served as protective factors for GD incidence. For the persistence of GD, higher education was protective, whereas higher perceived stress and gaming times served as risk factors.

### GD trends during the pandemic

Representative trends of GD in this study suggest an increase in prevalence during the COVID-19 pandemic, which aligned with other studies [16, 45, 46]. According to a systematic review, estimates increased during the pandemic up to 29.4% [16], possibly associated with school closures, isolation, increased stress, and future-related worries [47]. Additionally, given the lack of alternative activities and the shift to online schooling during the pandemic, parents may have been less restrictive with their children’s media rules, leading to an increased risk for problematic gaming among young adolescents.

Current prevalence rates in our study were higher compared to prepandemic levels. A growing trend in GD prevalence has also been described in other studies, particularly in Asia [48, 49]. However, the increase in GD may also reflect developments in the gaming industry over the past 5 years, including increased offers and device availability, earlier access among adolescents, optimized personalization, and the emergence of virtual and augmented reality, further fostering gaming immersion. Given that a substantial proportion of adolescents exhibit GD symptomatology, routine screening in pediatrics and child and adolescent psychiatry may help identify clinically relevant cases at an early stage. This is particularly important since a significant proportion of adolescents exhibiting GD symptomatology show comorbid psychiatric disorders such as depression [50], attention deficit hyperactivity disorder [51], or anxiety [52]. A recent longitudinal study suggests that psychopathology predicts GD symptoms [30], indicating that some children who seek treatment for anxiety or depression may also suffer from GD, which can, in turn, worsen their psychopathology.

### Sex- and age-specific GD trends during the pandemic

Interestingly, GD prevalence among females doubled in 2020 and reached identical values to males in 2022. These findings suggest that females are not inherently protected against the development of GD. Rather, external factors, such as the COVID-19 pandemic, may contribute to comparable prevalence estimates across sexes, despite females typically exhibiting lower values. Considering ongoing discussions on sex differences and the limited availability of data for females [26], these findings underscore the necessity of including both sexes in research to prevent the underrepresentation and inadequate treatment of either group. Interestingly, in contrast to males, the prevalence among females decreased steadily, potentially reflecting increasing engagement in alternative activities as life returned to normal. Only in 2021, significant age group differences in GD prevalence estimates were observed, reflecting variation over time.

### Incidence and persistence of GD development during the pandemic

The incidence of GD increased during the COVID-19 pandemic. In line with previous research, potential risk factors for GD incidence identified in our study were male sex, next to longer gaming times, and higher perceived stress [23, 53], suggesting the need for early prevention among this group. Higher education and older age acted as protective factors. Young adolescents, compared to older adolescents, may be in a more critical developmental phase marked by the onset of puberty and neurodevelopmental immaturity [5, 53]. This, in turn, may increase their vulnerability to developing GD and psychopathology in general [54]. Thus, prevention and treatment options are essential but often unavailable due to limited treatment capacity and insufficient clinical staff, resulting in prolonged waiting times. Research on digital interventions to support treatment of digital media use disorders, including GD, is currently ongoing (e.g., [55]) and suggests promising effects [56, 57].

When it comes to game design, adolescents may be particularly vulnerable to manipulative mechanisms aiming to increase gaming time and preoccupation, further elevating the risk of developing GD [58]. Game developers should be held accountable to ensure child safety, for example, by limiting manipulative design features [59]. This should be considered in age recommendations to advise parents. Prevention efforts should begin in school to target those at risk, and educational programs for parents/caregivers might enhance digital parental self-efficacy to combat critical periods for young adolescents [60, 61].

The temporal stability of GD was relatively low across the present sample. This is in line with previous research highlighting large (spontaneous) remission rates in adolescents [22, 53, 62]. However, persistence rates of more than 20% reflect a clinically significant proportion of adolescents urgently needing effective treatment to prevent further chronification and enduring negative consequences. Associated risk factors for persistence were longer gaming times and higher stress perception. Increased gaming time is positively correlated with GD [12] and might further increase stress due to the neglect of daily duties. However, stress and emotion coping capabilities of adolescents with GD are often limited and focused on gaming as a dysfunctional strategy [31]. Interestingly, age and biological sex were not significantly associated with the persistence of GD, indicating that even though males do show a higher prevalence of GD, they do not persist significantly more than females. Again, this argues against a general biologically determined risk of GD but suggests other mechanisms (e.g., preferences, comorbidities, environmental factors) to be at play, warranting further insight.

### Strengths and limitations

This longitudinal study with large representative samples of German adolescents is the first to provide robust evidence on GD epidemiology across 6 years, including the COVID-19 pandemic. By including data ranging from early to late adolescence, key developmental aspects over multiple years could be accounted for. Population-based weights were used for nationally representative samples, improving the generalizability. Standardized assessment according to ICD-11 criteria was applied. However, the following limitations should be considered: in line with other large epidemiological studies, validated self-report instruments were used to assess GD. To complement the findings, further research applying clinical diagnostic interviews should be conducted since self-report is prone to socially acceptable answers and recall bias, thus may lead to different results compared to diagnostic interviews or objective assessment [63]. The PSS-4 showed poor reliability of the sum score; conclusions relating to PSS-4 sum scores need to be drawn cautiously. Future studies should use additional instruments to verify results. The longitudinal study included irregular intervals between waves of data collection (12 months vs 6 months); i.e., changes over time need to be interpreted with care. Future studies should use coherent intervals throughout.

## Conclusion

This 6-year population-based longitudinal study supports GD to be a clinically relevant disorder among adolescents. Post-pandemic, adolescents continue to develop GD at higher rates, and a significant portion is currently in need of treatment. Early screening, specific prevention, and intervention programs are needed to target particularly vulnerable groups, including males, younger adolescents, lower education groups, and those with increased psychological stress and gaming time. The findings highlight the need for effective clinical treatment options and policy responses to counteract the negative impact of pathological gaming on children and youth.

## Supporting information

10.1192/j.eurpsy.2026.12232.sm001 Busch et al. supplementary material Busch et al. supplementary material

## Data Availability

The data supporting the findings of this study are part of an ongoing large study on problematic media use. They are available from the corresponding author upon reasonable request once all results have been published.

## Supplementary material

The supplementary material for this article can be found at http://doi.org/10.1192/j.eurpsy.2026.12232.

## Long descriptions

### Table 1. Long description

The table presents sociodemographic data across seven columns: Variables, 2019 (n = 1,206), 2020 (n = 1,128), 2021 (n = 1,103), 2022 (n = 1,010), 2023 (n = 1,067), and 2024 (n = 997). Data is reported as percentages or Mean (S D).

* Sex: The proportion of Females ranged from 47.9% to 48.9%; Males ranged from 51.1% to 52.1%.

* Age: The mean age remained stable around 13.5 years. The 10 to 13 years group represented approximately 48.7% to 50.3%, while the 14 to 17 years group represented 49.7% to 51.4%.

* Education: High education levels were most prevalent, with the largest proportion at 53.7% in 2024. Low education levels fluctuated between 9.5% and 15.9%.

* Place of residence: Urban residency was dominant, with 84.0% in 2019 and 79.9% in 2024. Rural residency ranged from 16.0% to 20.1%.

* Stress level (P SS-4 total score): Mean scores ranged from 5.3 to 6.3.

* Average weekly gaming time: Mean hours per week fluctuated between 11.8 and 13.8.

* GADIS-A total score: Mean scores ranged from 6.7 to 8.0.

Navigate back to Table 1..

### Table 2. Long description

The table presents prevalence estimates of GD as percentages with 95 percent CI in brackets across six years.

* Header Row: Columns for 2019 (n = 1,206), 2020 (n = 1,128), 2021 (n = 1,103), 2022 (n = 1,010), 2023 (n = 1,067), and 2024 (n = 997).

* Total Prevalence: Increases from 2.92 percent in 2019 to a peak of 6.53 percent in 2023, before dropping to 3.91 percent in 2024.

* Females:

- Total Females: Starts at 1.74 percent (2019), peaks at 6.13 percent (2022), and ends at 2.50 percent (2024).

- 10 to 13 years: Fluctuates from 1.92 percent (2019) to 6.74 percent (2021) and 3.22 percent (2024).

- 14 to 17 years: Starts at 1.56 percent (2019), peaks at 7.46 percent (2022), and ends at 1.78 percent (2024).

* Males:

- Total Males: Generally higher than females, starting at 4.03 percent (2019), peaking at 7.99 percent (2023), and ending at 5.23 percent (2024).

- 10 to 13 years: Increases from 2.85 percent (2019) to 7.67 percent (2023), then drops to 3.69 percent (2024).

- 14 to 17 years: Starts at 5.13 percent (2019), peaks at 8.30 percent (2023), and ends at 6.74 percent (2024).

Navigate back to Table 2..

### Figure 1. Long description

The figure consists of four panels (A, B, C, D) sharing a common X-axis for Year (2019 to 2024) and Y-axis for GD Prevalence percentage (0 to 12.5). Vertical red dashed lines are positioned at early 2020 and early 2022.

* Panel A: Overall GD prevalence. A black line with a grey 95 percent confidence interval ribbon shows a steady increase from approximately 3 percent in 2019 to a peak of 6.5 percent in 2023, followed by a sharp decline to 4 percent in 2024.

* Panel B: Prevalence by biological sex. An orange line (females) and a green line (males) both show upward trends. The male prevalence is consistently higher, peaking at 8 percent in 2023 before dropping to 5 percent in 2024. The female prevalence peaks at 6 percent in 2022 before declining to 2.5 percent in 2024.

* Panel C: Female prevalence by age. A solid orange line (10 to 13 years) shows a peak in 2021 at 6.5 percent, while a dotted orange line (14 to 17 years) shows a later peak in 2022 at 7.5 percent. Both age groups decline by 2024.

* Panel D: Male prevalence by age. A solid green line (10 to 13 years) and a dotted green line (14 to 17 years) show fluctuating but generally increasing trends until 2023. The 14 to 17 age group peaks higher at over 8 percent in 2023. Both groups show a decline in 2024.

Navigate back to Figure 1..

### Table 3. Long description

The table consists of three columns: Wave pair, between-wave Persistence of GD (Estimate percent and 95 percent CI), and between-wave Incidence of GD (Estimate percent and 95 percent CI).

* Wave pair W1 to W2: Persistence is 36.24 [12.25 to 60.22]; Incidence is 1.69 [0.66 to 2.72].

* Wave pair W2 to W3: Persistence is 31.30 [5.97 to 56.64]; Incidence is 3.44 [2.11 to 4.78].

* Wave pair W3 to W4: Persistence is 31.87 [15.47 to 48.26]; Incidence is 3.89 [2.46 to 5.33].

* Wave pair W4 to W5: Persistence is 31.53 [17.24 to 45.81]; Incidence is 3.56 [1.99 to 5.13].

* Wave pair W5 to W6: Persistence is 22.70 [8.14 to 37.25]; Incidence is 2.60 [1.32 to 3.88].

Note: Estimates are based on pooled results across 10 imputed datasets using Rubin's rules. GD stands for gaming disorder and CI stands for confidence interval.

Navigate back to Table 3..

### Table 4. Long description

The table contains five columns: Predictor, Incidence of GD OR [95% CI], p-values, Persistence of GD OR [95% CI], and p-values.

Key findings include:

* Age: Incidence OR 0.76, p .035; Persistence OR 0.88, p = .467.

* Biological sex (Reference: Females): Males show Incidence OR 1.78, p = .017; Persistence OR 1.44, p =.448.

* Education (Reference: Low): High education shows Incidence OR 0.40, p = .007 and Persistence OR .27, p = 0.01.

* Residence (Reference: Rural): Urban shows Incidence OR 1.32, p = .327; Persistence OR 0.79, p =.526.

* Covariates:

- Perceived stress: Incidence OR 1.41, p = .002; Persistence OR 1.86, p = .009.

- Gaming time: Incidence OR 1.37, p = less than .001; Persistence OR 1.63, p = less than .001.

* Start wave (Reference: Wave 1):

- Wave 2: Incidence OR 2.29, p = .034; Persistence OR 0.66, p = .515.

- Wave 3: Incidence OR 2.61, p = .01; Persistence OR 1.65, p= .319.

- Wave 4: Incidence OR 2.23, p = .041; Persistence OR 2.63, p = .057.

- Wave 5: Incidence OR 1.82, p = .152; Persistence OR 1.47, p = .513.

Note: Numeric variables were z-standardized. n = 3354 observations.

Navigate back to Table 4..
