# Supplementary material for: Epidemiology of adolescent gaming disorder: A 6-year population-based longitudinal study (2019–2024)
Source: Eur Psychiatry. 2026 Jun 15;69(1):e70. doi: 10.1192/j.eurpsy.2026.12232 (PMC13359008; doi:10.1192/j.eurpsy.2026.12232)
Supplement: Busch et al. supplementary material [file S0924933826122329sup001.pdf]

Epidemiology of Adolescent Gaming Disorder: A Six-Year Population-Based Longitudinal Study (2019-2024)

Katharina Busch[1], Hanna Wiedemann[1], Lisa Klamert[1], & Kerstin Paschke[1]

[1] German Center for Addiction Research in Childhood and Adolescence (DZSKJ), University Medical Center Hamburg-Eppendorf (UKE), Martinistrasse 52, D-20246 Hamburg, Germany

Contents

Figure S1. Flow chart of the population-based longitudinal study

Table S1. QIC to find the best-suited working correlation structure for GEE analyses

Table S2. Sensitivity analyses for GEE models using different working structure correlations – GD incidence

Table S3. Sensitivity analyses for GEE models using different working structure correlations – GD persistence

Table S4. Sociodemographic characteristics of German adolescents [representative data, non-imputed]

Table S5. Sociodemographic characteristics of German adolescents [wave-pair data]

Table S6. Sensitivity analyses (complete-case analysis) for GD prevalence estimates

Table S7. Pairwise comparisons for GD prevalence estimates stratified by sex and age group within each wave

Supplementary Methods.

Figure S1. Flow chart of the population-based longitudinal study.

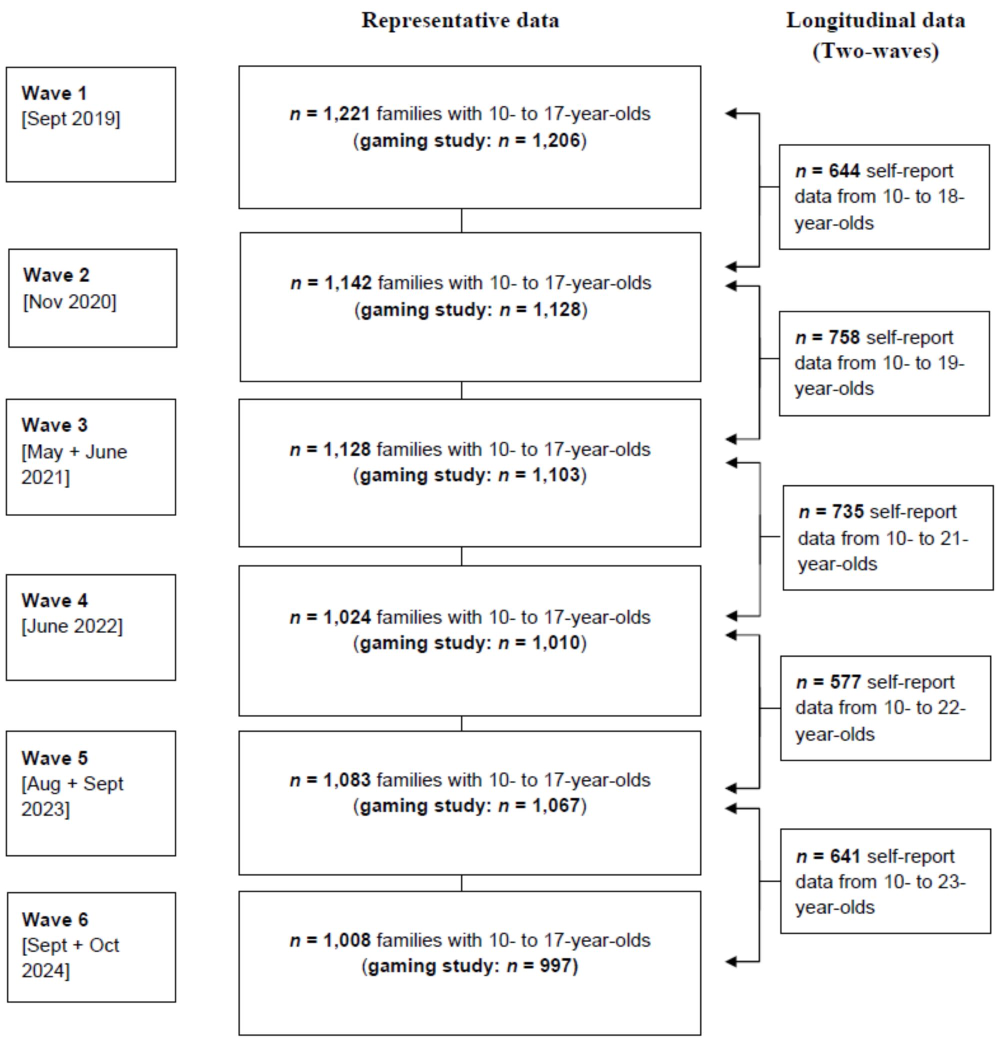

Table S1. QIC to find the best-suited working correlation structure for GEE analyses.

**Model:<br>Incidence of GD**	**Structure**	**QIC**	**Model:<br>Persistence of GD**	**Structure**	**QIC**
Model 1	Independence	822	Model 1	Independence	411
Model 2	Exchangeable	823	Model 2	Exchangeable	408
Model 3	AR(1)	822	Model 3	AR(1)	408

Note. Abbreviations: QIC Quasi-likelihood under the Independence model Criterion, GEE Generalized Estimating Equations, GD Gaming Disorder, AR(1) First-order autoregressive model.

Table S2. Sensitivity analyses for GEE models with different working correlation structures.

Model 1: Incidence of GD

Working correlation	Predictor	OR	CI (low)	CI (high)	*p*
**exchangeable**	**Age**	0.76	0.61	0.94	**.011**
	**Biological sex**				
	(Ref: Females)	—	—	—	—
	Males	1.78	1.13	2.79	**.012**
	**Education**				
	(Ref: Low)	—	—	—	—
	Middle	0.93	0.51	1.67	.801
	High	0.40	0.21	0.77	**.007**
	**Residence**				
	(Ref: Rural)	—	—	—	—
	Urban	1.32	0.75	2.33	.327
	**Start wave**				
	Wave 2	2.29	1.07	4.90	.034
	Wave 3	2.61	1.26	5.38	.01
	Wave 4	2.23	1.03	4.82	.041
	Wave 5	1.82	0.80	4.15	.152
	**Perceived Stress**	1.41	1.14	1.75	**.002**
	**Gaming Time**	1.37	1.20	1.56	**<.001**
**independence**	**Age**	0.75	0.61	0.94	**.011**
	**Biological sex**				
	(Ref: Females)	—	—	—	—
	Males	1.76	1.12	2.77	**.014**
	**Education**				
	(Ref: Low)	—	—	—	—
	Middle	0.92	0.51	1.65	.769
	High	0.40	0.20	0.77	**.006**
	**Residence**				
	(Ref: Rural)	—	—	—	—
	Urban	1.33	0.76	2.33	.322
	**Start wave**				

Working correlation	Predictor	OR	CI (low)	CI (high)	*p*
	Wave 2	2.29	1.06	4.94	.034
	Wave 3	2.64	1.27	5.46	.009
	Wave 4	2.29	1.06	4.94	.036
	Wave 5	1.86	0.82	4.23	.14
	**Perceived Stress**	1.42	1.15	1.76	**.001**
	**Gaming Time**	1.38	1.21	1.57	**<.001**
**AR(1)**	**Age**	0.75	0.60	0.93	**.009**
	**Biological sex**				
	(Ref: Females)	—	—	—	—
	Males	1.74	1.11	2.74	**.016**
	**Education**				
	(Ref: Low)	—	—	—	—
	Middle	0.90	0.50	1.63	.733
	High	0.40	0.20	0.77	**.006**
	**Residence**				
	(Ref: Rural)	—	—	—	—
	Urban	1.34	0.76	2.36	.304
	**Start wave**				
	Wave 2	2.27	1.05	4.91	.037
	Wave 3	2.67	1.29	5.53	.008
	Wave 4	2.38	1.11	5.10	.026
	Wave 5	1.87	0.82	4.25	.137
	**Perceived Stress**	1.44	1.17	1.78	**<.001**
	**Gaming Time**	1.40	1.23	1.59	**<.001**

Note. GEE Generalized Estimating Equations, GD Gaming Disorder, AR(1) First-order autoregressive model.

Table S3. Sensitivity analyses with different working correlation structures.

Model 2: Persistence of GD

**Working correlation**	**Predictor**	**OR**	**CI (low)**	**CI (high)**	***p***
	**Age**	0.88	0.63	1.24	.467
	**Biological sex**				
	(Ref: Females)	—	—	—	—
	Males	1.44	0.56	3.66	.448
	**Education**				
	(Ref: Low)	—	—	—	—
	Middle	0.73	0.31	1.76	.485
	High	0.27	0.10	0.73	**.01**
**exchangeable**	**Residence**				
	(Ref: Rural)	—	—	—	—
	Urban	0.79	0.31	2.01	.626
	**Start wave**				
	Wave 2	0.66	0.19	2.29	.515
	Wave 3	1.65	0.62	4.41	.319
	Wave 4	2.63	0.97	7.15	.057
	Wave 5	1.47	0.46	4.63	.513
	**Perceived Stress**	1.86	1.42	2.44	**<.001**
	**Gaming Time**	1.63	1.33	1.99	**<.001**
	**Age**	0.85	0.60	1.20	.351
	**Biological sex**				
	(Ref: Females)	—	—	—	—
	Males	1.43	0.55	3.70	.467
	**Education**				
**independence**	(Ref: Low)				
	Middle	0.73	0.30	1.80	.494
	High	0.25	0.09	0.71	**.009**
	**Residence**				
	(Ref: Rural)	—	—	—	—
	Urban	0.77	0.30	1.99	.592

**Working correlation**	**Predictor**	**OR**	**CI (low)**	**CI (high)**	***p***
	**Start wave**				
	Wave 2	0.58	0.13	2.50	.466
	Wave 3	1.77	0.61	5.15	.291
	Wave 4	2.89	0.99	8.45	.053
	Wave 5	1.69	0.51	5.54	.388
	**Perceived Stress**	1.98	1.51	2.58	**<.001**
	**Gaming Time**	1.66	1.35	2.04	**<.001**
	**Age**	0.90	0.64	1.27	.554
	**Biological sex**				
	(Ref: Females)	—	—	—	—
	Males	1.51	0.59	3.81	.388
	**Education**				
	(Ref: Low)	—	—	—	—
	Middle	0.72	0.30	1.70	.449
	High	0.27	0.10	0.74	**.011**
	**Residence**				
	(Ref: Rural)	—	—	—	—
	Urban	0.80	0.32	2.02	.636
	**Start wave**				
**AR(1)**	Wave 2	0.70	0.21	2.35	.559
	Wave 3	1.68	0.59	4.76	.333
	Wave 4	2.74	0.96	7.83	.06
	Wave 5	1.47	0.44	4.93	.535
	**Perceived Stress**	1.83	1.38	2.43	**<.001**
	**Gaming Time**	1.60	1.31	1.94	**<.001**

Note. GEE Generalized Estimating Equations, GD Gaming Disorder, AR(1) First-order autoregressive model.

Table S4. Sociodemographic characteristics of German adolescents [representative data, non-imputed]

Variables	**2019** *n* = 1,206 % / M (SD)	**2020** *n* =1,128 % / M (SD)	**2021** *n* = 1,103 % / M (SD)	**2022** *n* = 1,010 % / M (SD)	**2023** *n* = 1,067 % / M (SD)	**2024** *n* = 997 % / M (SD)
**Sex**						
Female	48.1	48.0	47.9	48.9	48.6	48.4
Male	52.9	52.0	52.1	51.1	51.4	51.6
**Age**	13.6 (2.3)	13.5 (2.2)	13.5 (2.3)	13.5 (2.2)	13.6 (2.3)	13.5 (2.3)
10-13 years	48.7	49.0	49.1	50.3	49.3	49.7
14-17 years	51.4	51.0	50.9	49.7	50.7	50.3
**Education**						
Low	9.6	10.8	11.7	13.7	15.8	11.8
Medium	37.6	37.1	38.9	39.1	31.3	34.6
High	52.8	52.1	49.4	47.2	52.9	53.6
NA	43	110	23	6	10	19
**Place of residence**						
Urban	84	83.0	81.4	79.4	79.5	79.9
Rural	16	17.0	18.6	20.6	20.6	20.1
**Stresslevel**						
PSS-4 total score	6.3 (2.9)	5.7 (3.0)	6.1 (2.9)	5.6 (3.1)	5.3 (2.8)	5.2 (2.7)
NA	159	85	81	83	101	61
**Average weekly gaming time (hours/week)**[a]	12.3 (14.2)	13.8 (15.3)	12.4 (13.3)	12.4 (13.1)	12.0 (11.8)	12.8 (14.8)
NA	64	55	98	105	87	108
**GADIS-A total score**	7.1 (7.2)	6.6 (7.6)	6.4 (7.3)	7.9 (8.1)	7.1 (8.1)	7.0 (7.6)
NA	51	21	48	20	22	26

Note. Based on weighted, non-imputed data.
[a]Based on regular users only (i.e., reported to game at least once per week).
Abbreviations: M mean, SD standard deviation, *n* = sample size, PSS-4 Perceived stress scale – short form, GADIS-A Gaming Disorder Scale for Adolescents.

Table S5. Sociodemographic characteristics of German adolescents [wave-pair data]

	2019-2020	2020-2021	2021-2022	2022-2023	2023-2024
	n = 644	*n* = 758	*n* = 735	*n* = 577	*n* = 641
**Variables**	%/ M (SD)	%/ M (SD)	%/ M (SD)	%/ M (SD)	%/ M (SD)
**Sex**					
Girls	46.7	47.0	48.8	47.4	46.2
Boys	53.3	53.0	51.2	52.6	52.8
**Age**	13.1 (2.4)	14.0 (2.5)	14.0 (2.6)	14.2 (2.7)	14.3 (2.8)
10-13 years	57.1	45.1	47.9	45.3	42.0
14-17 years	42.9	45.5	41.9	41.5	44.3
18-23 years	0	9.4	10.2	13.2	13.7
**Education**					
Low	8.7	8.9	7.81	12.7	11.8
Medium	35.0	34.2	37.1	36.2	34.6
High	56.3	56.9	55.1	51.1	53.6
**Residence**					
Urban	83.1	82.6	79.7	80.7	18.9
Rural	16.9	17.4	20.3	19.3	81.1
**Stresslevel**					
PSS-4 total score	6.04 (2.9)	5.39 (3.0)	6.01 (2.9)	5.42 (3.1)	5.2 (3.1)
**Average weekly gaming time (hours/week)**[a]	10.9 (12.3)	12.6 (14.4)	11.8 (11.7)	12.9 (13.7)	11.7 (11.7)
**GADIS-A total score**	6.72 (6.9)	6.25 (6.9)	6.83 (7.4)	8.17 (8.3)	6.93 (7.8)

Note. Multiply imputed data. Based on baseline values (first wave) of each two-wave period.

[a]Based on regular users only (i.e., reported to game at least once per week).

Abbreviations: M mean, SD standard deviation, *n* = sample size, PSS-4 Perceived stress scale – short form, GADIS-A Gaming Disorder Scale for Adolescents.

Table S6. Sensitivity analyses (complete-case analysis) using non-imputed data for GD prevalence estimates.

	**2019** *n* = 1,206		**2020** *n* =1,128		**2021** *n* = 1,103	
	Estimate % [95 % CI]		Estimate % [95 % CI]		Estimate % [95 % CI]	
**Groups**	**non-imputed + weighted**	**imputed + weighted**	**non-imputed + weighted**	**imputed + weighted**	**non-imputed + weighted**	**imputed + weighted**
**Total**	2.67 [1.73;4.10]	2.92 [1.90;3.95]	3.63 [2.26;5.77]	3.86 [2.59;5.13]	4.38 [2.80;6.79]	5.15 [3.77;6.53]
**Females**	1.79 [0.80;3.95]	1.74 [0.67;2.8]	3.32 [1.68;6.44]	3.36 [1.79;4.92]	3.00 [1.05;8.29]	3.62 [2.02;5.21]
10-13 years	1.99 [0.83;4.72]	1.92 [0.32;3.51]	4.59 [2.12;9.67]	4.45 [1.97;6.93]	5.68 [1.81;16.47]	6.74 [3.70;9.79]
14-17 years	1.59 [0.38;6.39]	1.56 [0.15;2.97]	2.12 [0.55;7.78]	2.31 [0.38;4.24]	0.56 [0.16;1.99]	0.62 [-0.37;1.60]
**Males**	3.51 [2.09;5.82]	4.03 [2.32;5.73]	3.91 [2.03;7.41]	4.33 [2.42;6.24]	5.65 [3.66;8.62]	6.55 [4.38;8.73]
10-13 years	1.98 [0.92;4.19]	2.85 [0.57;5.13]	3.66 [1.11;11.38]	3.95 [1.55;6.35]	6.00 [3.44;10.25]	5.92 [3.15;8.68]
14-17 years	4.93 [2.56;9.28]	5.13 [2.65;7.62]	4.15 [2.06;8.17]	4.70 [1.87;7.53]	5.31 [2.68;10.27]	7.17 [3.83;10.5]

Note. Abbreviations: CI Confidence Interval, GD Gaming Disorder, *n* = sample size. Non-imputed data refers to observed data only (i.e., complete-case analysis). Imputed + weighted refers to weighted prevalence estimates based on pooled results across multiple imputed datasets (m = 10).

Table S6. Sensitivity analyses (complete-case analysis) using non-imputed data for GD prevalence estimates (continued).

	**2022** *n* = 1,010		**2023** *n* = 1,067		**2024** *n* = 997	
	Estimate % [95 % CI]		Estimate % [95 % CI]		Estimate % [95 % CI]	
**Groups**	**non-imputed + weighted**	**imputed + weighted**	**non-imputed + weighted**	**imputed + weighted**	**non-imputed + weighted**	**imputed + weighted**
**Total**	6.05 [4.28;8.49]	6.13 [4.64;7.61]	6.55 [4.70;9.05]	6.53 [5.03;8.03]	3.66 [2.31;5.77]	3.91 [2.70;5.12]

**Females**	5.95 [3.36;10.32]	6.13 [4.01;8.24]	5.04 [2.55;9.71]	4.99 [3.11;6.87]	2.54 [1.01;6.21]	2.50 [1.10;3.89]
10-13 years	4.28 [1.59;11.03]	4.79 [2.12;7.46]	6.42 [2.42;15.98]	6.35 [3.35;9.35]	3.23 [1.16;8.66]	3.22 [0.98;5.45]
14-17 years	7.62 [3.76;14.83]	7.46 [4.18;10.74]	3.71 [1.65;8.10]	3.67 [1.40;5.95]	1.83 [0.30;10.42]	1.78 [0.11;3.45]
**Males**	6.15 [4.08;9.18]	6.13 [4.05;8.21]	8.00 [5.67;11.18]	7.99 [5.69;10.29]	4.75 [2.82;7.88]	5.23 [3.29;7.18]
10-13 years	6.60 [3.69;11.54]	6.45 [3.47;9.44]	8.24 [5.04;13.19]	7.67 [4.45;10.89]	3.48 [1.75;6.80]	3.69 [1.34;6.05]
14-17 years	5.70 [3.17;10.03]	5.80 [2.91;8.68]	8.03 [4.91;12.86]	8.30 [5.03;11.57]	6.04 [2.92;12.05]	6.74 [3.68;9.80]

Note. Abbreviations: CI Confidence Interval, GD Gaming Disorder, *n* = sample size. Non-imputed data refers to observed data only (i.e., complete-case analysis). Imputed + weighted refers to weighted prevalence estimates based on pooled results across multiple imputed datasets (m = 10).

Table S7. Pairwise comparisons for GD prevalence estimates stratified by sex and age group within each wave.

Year	Comparison	Δ (%)	95% CI	z	*p-uncorrected*	*p-corrected*
2019	Females vs. Males	2.3	[0.3; 4.3]	2.234	**.025***	**.075†**
	Females aged 14-17 years vs. Females aged 10-13 years	-0.4	[-2.5; 1.8]	-0.327	.743	.743
	Males aged 14-17 years vs. Males aged 10-13 years	2.3	[-1.1; 5.6]	1.341	.180	.270
2020	Females vs. Males	1	[-1.4; 3.4]	0.789	.430	.645
	Females aged 14-17 years vs. Females aged 10-13 years	-2.1	[-5.3; 1]	-1.332	.183	.549
	Males aged 14-17 years vs. Males aged 10-13 years	0.8	[-2.9; 4.4]	0.409	.683	.683
2021	Females vs. Males	2.9	[0.3; 5.6]	2.145	**.032***	**.048***
	Females aged 14-17 years vs. Females aged 10-13 years	-6.1	[-9.3; -2.9]	-3.751	**.0002*****	**.0006*****
	Males aged 14-17 years vs. Males aged 10-13 years	1.2	[-3.1; 5.6]	0.565	.572	.572
2022	Females vs. Males	0	[-3; 3]	0.002	.998	.998
	Females aged 14-17 years vs. Females aged 10-13 years	2.7	[-1.6; 6.9]	1.239	.2152	.646
	Males aged 14-17 years vs. Males aged 10-13 years	-0.7	[-4.8; 3.5]	-0.309	.7571	.998
2023	Females vs. Males	3	[0; 6]	1.980	**.048***	.144
	Females aged 14-17 years vs. Females aged 10-13 years	-2.7	[-6.4; 1.1]	-1.394	.1634	.245
	Males aged 14-17 years vs. Males aged 10-13 years	0.6	[-3.9; 5.2]	0.267	.7893	.789
2024	Females vs. Males	2.7	[0.3; 5.1]	2.241	**.025***	**.075†**
	Females aged 14-17 years vs. Females aged 10-13 years	-1.4	[-4.2; 1.3]	-1.014	.3118	.312
	Males aged 14-17 years vs. Males aged 10-13 years	3.0	[-0.8; 6.9]	1.548	.1216	.182

Note. Multiply imputed data. Abbreviations: Δ (%) represents differences in predicted probabilities (percentage points), 95 % CI 95 % Confidence Interval, z z-statistic, *p-uncorrected* p-value without correction, *p-corrected* p-values corrected for multiple testing using False Discovery Rate (FDR). † $p < .10$, * $p < .05$, ** $p < .01$, *** $p < .001$.

Supplementary Methods.

The data used in this study is part of a larger study on **digital media use and mental health among German families** (i.e., adolescents and respective parents). It is an ongoing study since 2019 with yearly surveys collected by ***forsa***, an established German market research and opinion polling company.

Participants were recruited from the web-based forsa.Omninet panel by the German Institute for Social Research and Statistical Analysis, ***forsa*** [34]. The ***forsa*** database includes more than 100,000 German adults and adolescents. For each wave, participants were selected on the basis of all available representatives from a cluster of German adults aged 28 to 75 years. Respectively, approximately 15,000 representative households were contacted via email in each wave. Of the respondents, only those that reported having children between 10 and 17 years were included. Among households with more than one child between 10 and 17 years, surveys were only conducted with the minor whose birth date was the most recent at the time of the survey to ensure a balanced proportion of age groups and gender. Additionally, in each wave, participants from previous waves were invited to participate again, with some participants exceeding 17 years of age. A final number of participants (around 1,200 each year) provided necessary details and gave consent for participation. Please find more details on the recruitment of adolescents and parents for the study in **Paschke et al., 2021a** (Link to manuscript: https://doi.org/10.1192/bjo.2021.49) and **Paschke et al., 2021b** (Link to manuscript: https://doi.org/10.1556/2006.2020.00105).
